# Glucagon‐like peptide‐1 elicits vasodilation in adipose tissue and skeletal muscle in healthy men

**DOI:** 10.14814/phy2.13073

**Published:** 2017-02-08

**Authors:** Ali Asmar, Meena Asmar, Lene Simonsen, Sten Madsbad, Jens J Holst, Bolette Hartmann, Charlotte M Sorensen, Jens Bülow

**Affiliations:** ^1^ Department of Clinical Physiology and Nuclear MedicineBispebjerg University HospitalCopenhagenDenmark; ^2^ Department of EndocrinologyHvidovre University HospitalCopenhagenDenmark; ^3^ NNF Center for Basic Metabolic ResearchUniversity of CopenhagenCopenhagenDenmark; ^4^ Department of Biomedical SciencesUniversity of CopenhagenCopenhagenDenmark

**Keywords:** Adipose tissue, blood flow, GLP‐1, skeletal muscle, splanchnic circulation, vasodilation

## Abstract

In healthy subjects, we recently demonstrated that during acute administration of GLP‐1, cardiac output increased significantly, whereas renal blood flow remained constant. We therefore hypothesize that GLP‐1 induces vasodilation in other organs, for example, adipose tissue, skeletal muscle, and/or splanchnic tissues. Nine healthy men were examined twice in random order during a 2‐hour infusion of either GLP‐1 (1.5 pmol kg^−1^ min^−1^) or saline. Cardiac output was continuously estimated noninvasively concomitantly with measurement of intra‐arterial blood pressure. Subcutaneous, abdominal adipose tissue blood flow (ATBF) was measured by the ^133^Xenon clearance technique. Leg and splanchnic blood flow were measured by Fick's Principle, using indocyanine green as indicator. In the GLP‐1 study, cardiac output increased significantly together with a significant increase in arterial pulse pressure and heart rate compared with the saline study. Subcutaneous, abdominal ATBF and leg blood flow increased significantly during the GLP‐1 infusion compared with saline, whereas splanchnic blood flow response did not differ between the studies. We conclude that in healthy subjects, GLP‐1 increases cardiac output acutely due to a GLP‐1‐induced vasodilation in adipose tissue and skeletal muscle together with an increase in cardiac work.

## Introduction

We recently reported substantial acute effects of physiologically increased plasma levels of GLP‐1 on cardiovascular hemodynamics in humans, using continuous (invasive and noninvasive) measurements (Asmar et al. 2015, 2016a). In healthy individuals (Asmar et al. 2015) and patients with type 2 diabetes (Asmar et al. 2016a), we demonstrated a GLP‐1‐induced increase in heart rate, possibly due to direct effects of GLP‐1 on the heart (Pyke et al. 2014). Furthermore, GLP‐1 increased cardiac output in healthy subjects (~18%, 1.2 ± 0.1 L/min) but not in patients with type 2 diabetes. Despite a significant renal clearance of GLP‐1, exceeding glomerular filtration (~55%), renal blood flow and glomerular filtration rate remained unchanged. The increase in cardiac output was proportionally greater than the increase in mean arterial pressure (~2%, 2.9 ± 1.4 mmHg), suggesting a vasodilation in one or more vascular beds except for the renal vascular bed.

Recently (Koska et al. 2015), it has been demonstrated that GLP‐1 receptors are functionally expressed in the endothelium. GLP‐1 receptor engagement improves endothelial function in patients with type 2 diabetes. The improved endothelial function is probably via stimulation of endothelial AMP‐activated protein kinase pathway activity. This was demonstrated in isolated human adipose tissue arterioles, resulting in a greater eNOS activity, inducing vasodilation. However, the GLP‐1‐mediated vasodilation may also occur by mechanisms independent of the epithelium. Using a validated monoclonal antibody for immunohistochemistry, Pyke et al. (2014) detected vascular GLP‐1 receptors exclusively in the smooth muscle cells in arteries and arterioles. Engagement of the GLP‐1 receptor in vascular smooth muscle cells leads to cAMP formation and thereby activation of downstream paths involving protein kinase A (PKA) and exchange protein directly activated by cAMP (EPAC) (Drucker 2006). Both PKA and EPAC induce intracellular Ca^2+^ accumulation, initiating vascular relaxation via not fully clarified signaling pathways (Gloerich and Bos 2010; Leech et al. 2010).

Using the flow‐mediated dilation technique, Basu et al. (2007) demonstrated, in healthy subjects, that acute infusion of GLP‐1 increases forearm blood flow in response to acetylcholine, whereas Nystrom et al. (2004) demonstrated that acute administration of GLP‐1 increased forearm blood flow in type 2 diabetes patients with stable coronary artery disease, but not in healthy subjects. Using real‐time, contrast‐enhanced ultrasound technique, Sjoberg et al. (2014) demonstrated microvascular recruitment in the vastus lateralis muscle in healthy subjects during an acute administration of GLP‐1. Aside from this, it is not fully clarified whether GLP‐1, in vivo*,* modulates vascular tonus in other beds, for example, adipose tissue and/or splanchnic tissues.

Therefore, we designed the present randomized, placebo‐controlled, and single‐blinded experiment to elucidate whether acute administration of GLP‐1, under fixed sodium intake, induces vasodilation in adipose tissue, skeletal muscle, and/or splanchnic tissues in healthy subjects.

## Materials and Methods

## Subjects

Baseline characteristics are shown in Table 1. Nine lean young male subjects of Caucasian origin participated in the study, which involved two experiments performed in random order separated by about 4 weeks. All subjects were healthy and none took medication at the time of the study. Body composition was determined by dual energy X‐ray absorptiometry (DEXA) scanning (Lunar iDXA; GE Healthcare, Brøndby, Denmark) (Table 1). Consent to participate was obtained after the subjects had read a description of the experimental protocol, which was approved by the Scientific Ethics Committee of the capital region of Copenhagen (H‐1‐2014‐089).

**Table 1:** Baseline characteristics

Variable	Value
Age (years)	23 ± 2
Height (cm)	183 ± 3
Weight (kg)	78.0 ± 4.3
Lean body mass (kg)	60.0 ± 6.1
Whole body fat mass (kg)	14.8 ± 5.2
Right leg lean mass (kg)	10.6 ± 0.5
Right leg fat mass (kg)	2.6 ± 0.3
Systolic blood pressure (mmHg)	126 ± 9
Diastolic blood pressure (mmHg)	71 ± 10
Heart rate (bpm)	56 ± 9
Fasting glucose concentration (mmol/L)	5.4 ± 0.3
Fasting insulin concentration (pmol/L)	17.8 ± 15.1
Urinary albumin excretion (mg/24‐h)	2.2 ± 1.3
Urinary glucose excretion (mmol/24‐h)	0.4 ± 0.1

Body composition was determined by dual energy X‐ray absorptiometry (DEXA) scanning. Data are presented as mean ± SD.

One subject developed syncope during the catheterization procedures. Thus, only ^133^Xe washout data from this subject were included in further analyses.

### Protocol

**Figure 1:**
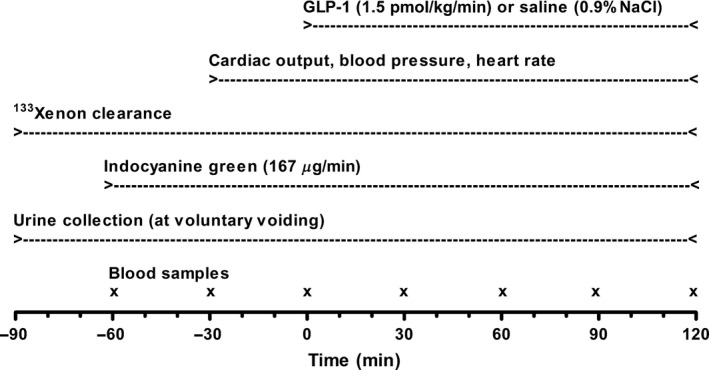
The experimental timeline.

For 4 days before each experiment all subjects consumed a controlled mixed diet (2822 kcal per day, 16% protein, 55% carbohydrate, 29% fat). The food was handed out frozen, and the basal sodium chloride content of the diet, measured at Eurofins Stein's Laboratory in Denmark, was 55‐75 mmol per day. Sodium chloride was added to the diet in order to standardize daily intake at 2 mmol sodium chloride per kg body weight per day (Asmar et al. 2015, 2016a). 24‐h urine was collected on the last day and electrolyte, albumin, and glucose concentrations were determined. Water intake was ad libitum, and strenuous excess physical activity was not allowed. Subjects fasted for 12 h before the beginning of the experiment. The experimental timeline is shown in Figure 1. After emptying the bladder, confirmed by ultrasound, subjects remained supine throughout the experiments. During the experiments, bladder emptying was allowed with subjects remaining in the supine position.

### Catheterization

A forearm vein was catheterized with an 18 gauge catheter (BD Venflon^®^: length 45 mm, Becton Dickinson, Helsingborg, Sweden) for infusions. The right femoral vein was catheterized using the Seldinger technique and a 7 F introducer (Radifocus^®^: length 110 mm, Terumo Corporation, Leuven, Belgium). A 6 F catheter (Check‐Flo Performer^®^: length 750 mm, Cook Medical, Bloomington, IN) was advanced into a right‐sided hepatic vein under fluoroscopic control. The right femoral artery was catheterized, using the Seldinger technique and a 3 F catheter (Femoral Artery Catheter Set: length 80 mm, Cook Medical, Bloomington, IN) for continuous infusion of indocyanine green (ICG). In the nondominant arm, the radial artery was catheterized with a 20 gauge catheter (BD Arterial Cannula^®^: length 45 mm, Becton Dickinson Medical, Franklin Lakes, NJ) for blood sampling and for continuous monitoring of arterial blood pressure. Blood was collected simultaneously from the radial artery and the femoral and hepatic vein throughout the experiments as described below.

### Blood flow measurements

Subcutaneous, abdominal ATBF was calculated from the washout rate constant of ^133^Xenon. This technique has previously been validated in our laboratory (Simonsen et al. 2003). About 1.0 MBq gaseous ^133^Xenon mixed in about 0.1 ml atmospheric air was injected into the para‐umbilical area of the subcutaneous adipose tissue. The washout rate of ^133^Xenon was measured continuously by a scintillation counter system (Oakfield Instruments, Oxford, UK) strapped to the skin surface above the ^133^Xenon depot. Measurements obtained during periods of 20 min throughout infusions were used for analyses. Leg and splanchnic blood flow were measured via Fick's Principle, using ICG as indicator as previously described (Enevoldsen et al. 2004; Hovind et al. 2010). An intravenous bolus injection of ICG (1 mg) in 10 mL of 0.9% NaCl was administered followed by a continuous intra‐arterial infusion (167 *μ*g min^−1^) in 0.9% NaCl (70 mL h^−1^). Steady state arterial concentrations of ICG were obtained after ~60 min. After at least 60 min of infusion of ICG and after a stable monoexponential washout of ^133^Xenon was registered, two baseline blood sample pairs were drawn followed by start of a 2‐h infusion of either GLP‐1 (1.5 pmol kg^−1^ min^−1^) or saline (0.9% NaCl). The solutions were prepared freshly. The subjects were blinded with respect to the contents.

### Central hemodynamics

Blood pressure and heart rate were monitored invasively (ADInstruments, Oxford, UK) throughout the experiments. Estimated cardiac output was recorded continuously and noninvasively using Finapres (Finapres Medical Systems BV, Amsterdam, The Netherlands) (Imholz et al. 1998). The estimation of cardiac output via pulse contour analysis is an indirect method based on the development of the pulsatile unloading of the finger arterial walls using an inflatable finger cuff with built‐in photo‐electric plethysmograph (Langewouters et al. 1984, 1985). To achieve highest accuracy and precision regarding absolute stroke volume levels and cardiac output levels, a calibration of the Finapres against a direct method such as the Fick's Principle (e.g., indicator‐dilution) is required. Such calibration is, however, not necessary to observe relative changes in cardiac output due to the GLP‐1 infusion (Stok et al. 1993; Bogert and van Lieshout 2005). Measurements obtained during periods of ~5 min before and after blood sampling were used for analyses.

### Blood and urine analyses

Samples of blood were drawn simultaneously from the radial artery and the right‐sided femoral and hepatic vein every 30 min from time −30 min until termination of the experiments (Fig. 1). All arterial as well as venous blood samples were analyzed for GLP‐1 and ICG. Glucose and insulin were analyzed only in arterial blood samples. The amount of collected blood was substituted with a similar amount of isotonic saline during the experiments.

Plasma samples were assayed for total GLP‐1 immunoreactivity and for intact GLP‐1, as previously described (Orskov et al. 1994; Wewer Albrechtsen et al. 2015). Concentrations of the primary metabolite GLP‐1 9‐36amide were calculated by subtraction of concentrations of intact GLP‐1 from total concentrations (Meier et al. 2004).

Plasma insulin levels were measured using a commercial enzyme immunoassay kit (Insulin Human ELISA EIA‐2935, AH Diagnostics, Aarhus, Denmark).

Blood glucose concentrations and hematocrit were measured using an automated benchtop blood analyzer system (ABL 700 series, Radiometer Medical Aps, Brønshøj, Denmark).

Plasma ICG concentrations were determined by spectrophotometry at 805 and 904 nm in duplicates as previously described (Enevoldsen et al. 2005).

Urinary electrolyte concentrations were measured by atomic absorption (Atomic absorption spectrophotometer model 2380, PerkinElmer, Norwalk, Connecticut). Urinary pH was measured using a XC161 Combination pH electrode (Radiometer Medical Aps, Brønshøj, Denmark). Urinary albumin and glucose concentrations were measured using an enzymatic method (Cobas Integra^®^ 400, Roche Diagnostics, Indianapolis, IN).

### Materials

Synthetic human GLP‐1 7–36amide was obtained from Clinalfa^®^, Bachem (Bubendorf, Switzerland), ICG from Pulsion Medical Systems (Feldkirchen, Germany), and ^133^Xenon from DRAXIMAGE^®^ (Québec, Canada).

### Calculations

The subcutaneous, abdominal ATBF was calculated from the mean ^133^Xenon washout rate constant determined in 20‐min periods. Thus, ATBF was calculated according to the equation ATBF = −k × *λ *× 100. A tissue/blood partition coefficient (*λ*) for Xenon of 10 mL g^−1^ was used (Bulow et al. 1987b).

Leg plasma flow was calculated as: ICG infusion‐rate/(ICG_femoral venous_ – ICG_arterial_) at steady state, and leg blood flow was subsequently calculated from simultaneous measurements of hematocrit.

Splanchnic plasma flow was calculated as: ICG infusion‐rate/(ICG_arterial_ – ICG_hepatic venous_) at steady state, and splanchnic blood flow was calculated on the basis of simultaneous measurements of hematocrit.

The extraction ratio of GLP‐1 in the lower extremity and splanchnic region was calculated as: (GLP‐1_arterial_‐GLP‐1_venous_)/GLP‐1_arterial_.

### Statistical analysis

The primary end‐point in this study was the cardiac output. When using a 2‐tailed *α *= 0.05 and requiring an 80% power threshold, the sample size *n* < 6 was calculated to detect an appreciable effect of GLP‐1 on cardiac output. This calculation was based on our previous study (Asmar et al. 2015), in which the effect magnitude of a 3‐h intravenous GLP‐1 infusion on cardiac output was 1.2 L min^−1^ with an SD of 0.2 L min^−1^.

Data were analyzed, using SigmaPlot 12 (Systat Software, Inc., Chicago, IL) and GraphPad Prism 5 (GraphPad Software, Inc., La Jolla, CA). Area under the curve (AUC) was calculated, using the trapezoidal rule, and the *t*‐test (2‐tailed) for paired data was used for comparing ΔAUC during the GLP‐1 infusion and ΔAUC during the saline infusion. Values of *P* < 0.05 were considered statistically significant.

## Results

### Standardized sodium chloride intake

On the last day of the 4‐day period with standardized sodium chloride intake prior to the GLP‐1 or saline study, 24‐h renal sodium excretions (data not shown) were equal between subjects. Using samples from urine collected throughout the GLP‐1 and saline studies (328 ± 5 min and 333 ± 9 min), mean urinary sodium, potassium and hydrogen excretions were not statistically different on the 2 days (data not shown).

### Capillary GLP‐1 degradation

**Figure 2:**
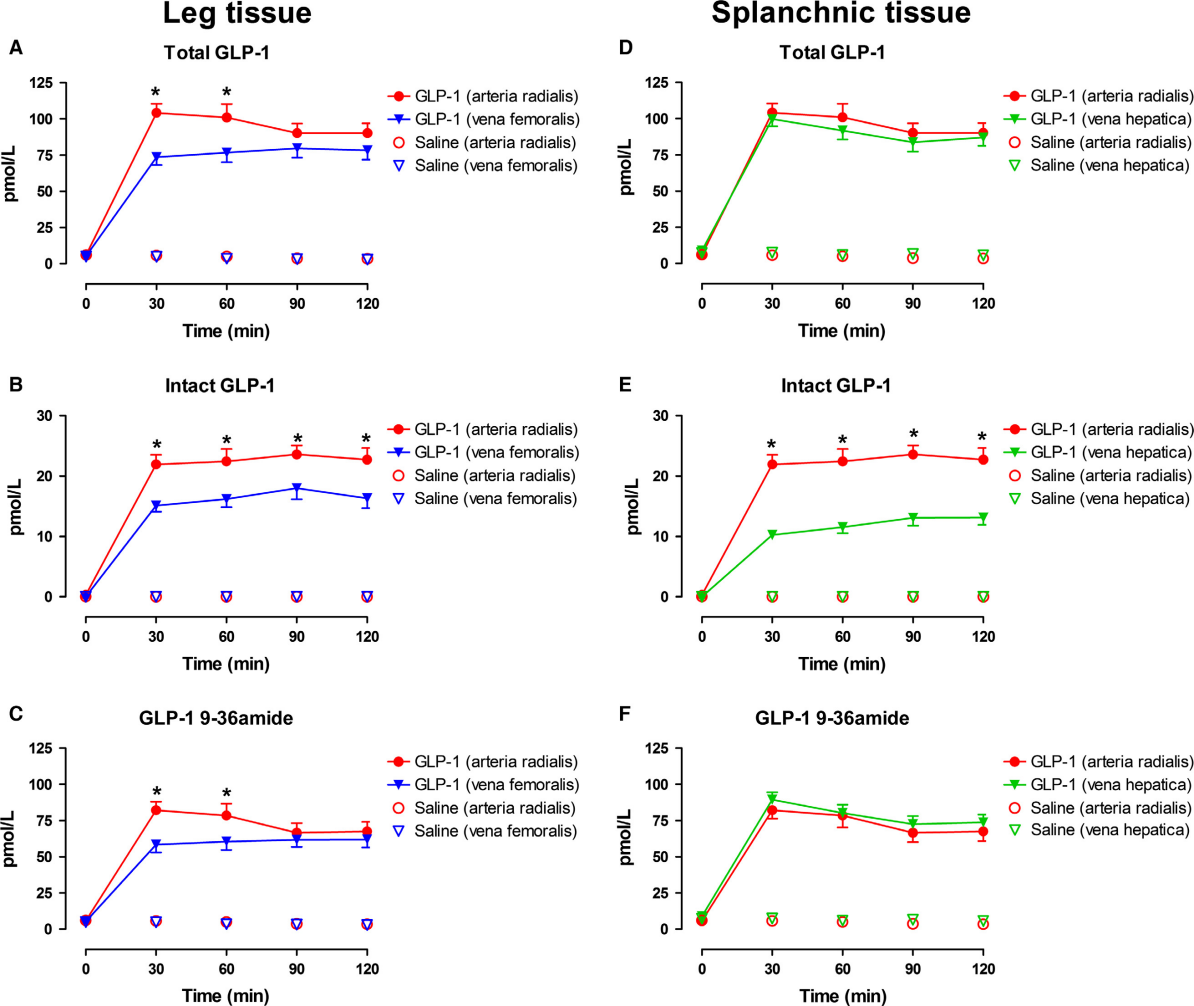
Arterial (radial) and venous (femoral and hepatic) plasma concentrations of total GLP‐1 (A and D), intact GLP‐1 (B and E), and GLP‐1 9‐36amide (C and F) during the GLP‐1 or saline infusion. Data are presented as means ± SE. *indicates statistically significant extraction of GLP‐1 during the GLP‐infusion compared with saline.

Lower extremity and splanchnic arteriovenous plasma levels of total GLP‐1, intact GLP‐1, and GLP‐1 9‐36amide during the GLP‐1 and saline infusions are shown in Figure 2.

During the GLP‐1 infusion, arterial plasma concentrations of total GLP‐1 increased significantly to 96 ± 7 pmol/L (Fig. 2A) and femoral as well as hepatic venous plasma concentrations increased significantly to 77 ± 6 pmol/L (Fig. 2A) and 91 ± 6 pmol/L (Fig. 2D), respectively. Analysis of the separate contributions of intact GLP‐1 7–36amide demonstrated a significant arteriovenous concentration difference (femoral, ~30%, Fig. 2B and hepatic, ~50%, Fig. 2E). The calculated GLP‐1 9‐36amide metabolite showed a smaller arteriovenous concentration difference (femoral, ~25%, *P* = 0.090, Fig. 2C), whereas no hepatic arteriovenous GLP‐1 9–36amide concentration difference could be demonstrated (Fig. 2F). During the saline infusion, arterial and venous plasma concentrations of total GLP‐1 remained constant throughout the experiments, and a significant arteriovenous concentration difference of GLP‐1 could not be demonstrated (Fig. 2A–F).

### Effects of GLP‐1 on arterial blood glucose and plasma insulin

**Figure 3:**
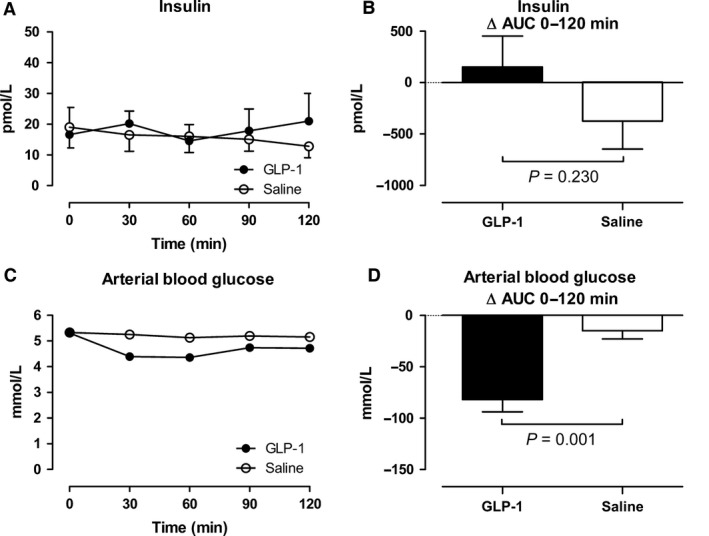
Arterial plasma concentrations of insulin (A and B) and arterial blood glucose concentrations (C and D). Left panel shows the time course of the concentrations during the infusions from 0 to 120 min. Right panel shows the integrated effect during the infusions from 0 to 120 min compared with baseline. Data are presented as means ± SE.

Arterial plasma insulin concentrations and arterial blood glucose concentrations during the GLP‐1 and saline infusions are shown in Figure 3.

During the GLP‐1 infusion, arterial plasma insulin concentrations tended to increase transiently from 16.6 pmol/L to 21.0 pmol/L, whereas an increase was not seen during the saline infusion (Fig. 3A and B). During the GLP‐1 infusion, arterial blood glucose concentrations were transiently reduced (*P* = 0.001) (Fig. 3C and D) with a nadir of 4.36 ± 0.12 mmol/L at 60 minutes, and with a range of 3.90–5.73 mmol/L within the first 60 min (Fig. 3C). None of the subjects developed symptoms of hypoglycemia. After a transient ~2‐fold reduction in splanchnic glucose output concomitant with the increase in insulin concentration 30 min after the commencement of the GLP‐1 infusion, the splanchnic glucose output returned to baseline level, ~1 mmol min^−1^, indicating that the hypoglycemia did not elicit significant metabolic counter regulation. During the saline infusion, blood glucose concentrations remained unchanged, and the splanchnic glucose output remained constant ~1 mmol min^−1^.

### Effects of GLP‐1 on central hemodynamics

**Figure 4:**
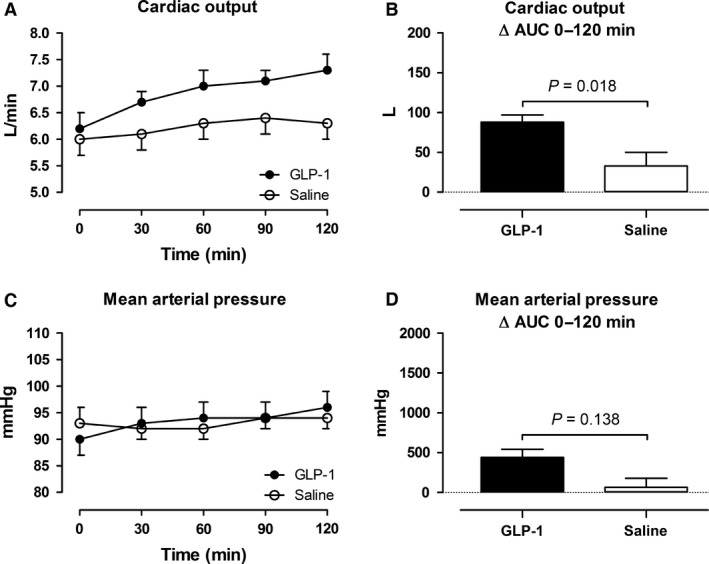
Cardiac output (A and B) and mean arterial pressure (C and D). Left panel shows the time course of the measurements during the infusions from 0 to 120 min. Right panel shows the integrated effect during the infusions from 0 to 120 min compared with baseline. Data are presented as means ± SE.

**Figure 5:**
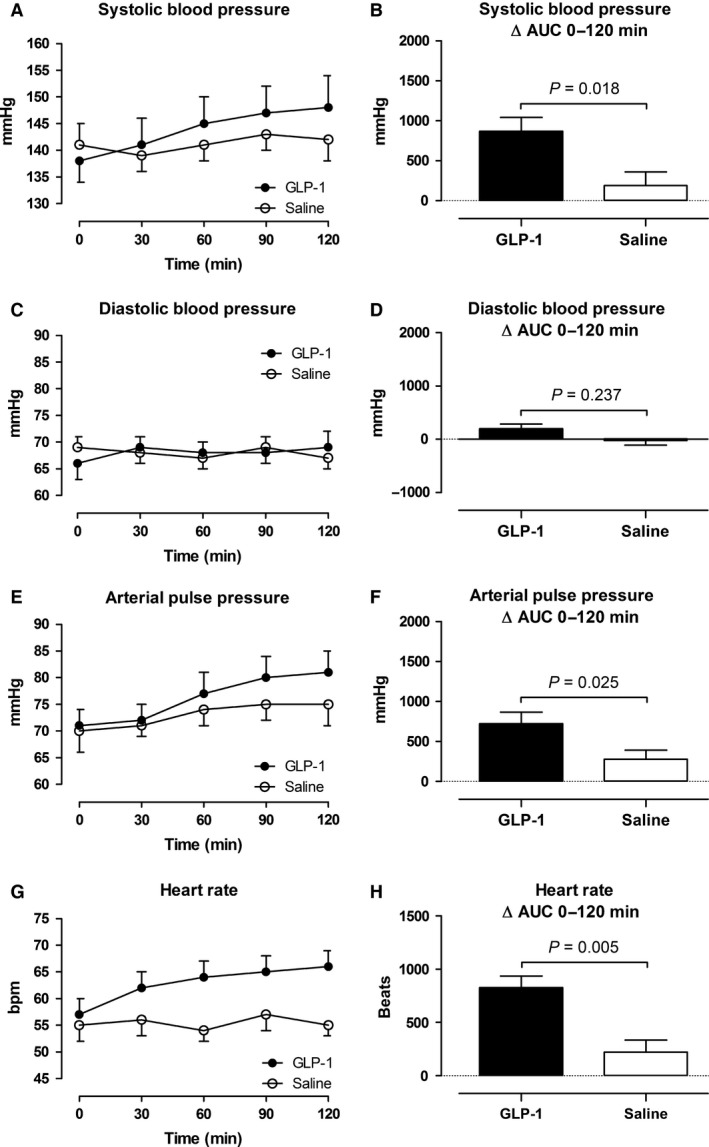
Intra‐arterial blood pressure (A–F) and heart rate (G and H). Left panel shows the time course of the measurements during the infusions from 0 to 120 min. Right panel shows the integrated effect during the infusions from 0 to 120 min compared with baseline. Data are presented as means ± SE.

Cardiac output, blood pressure and heart rate during the GLP‐1 and saline infusions are shown in Figure 4 and 5.

In the GLP‐1 study, cardiac output increased in average by 0.8 ± 0.1 L min^−1^ (13%) with a maximal increase (90–120 min after the commencement of the infusion) by 1.1 ± 0.1 L min^−1^ (18%). In the saline study, the peak increase (90–120 min after the commencement of the infusion) was 0.3 ± 0.1 L min^−1^ (Fig. 4A and B). Systolic blood pressure increased significantly by 7 ± 1 mmHg in the GLP‐1 study compared to an increase by 2 ± 1 mmHg in the saline study, (Fig. 5A and B). Diastolic blood pressure remained unchanged in both studies (Fig. 5C and D). Arterial pulse pressure increased significantly in the GLP‐1 study by 6 ± 1 compared to an increase by 3 ± 1 mmHg in the saline study (Fig. 5E and F). Mean arterial pressure tended to increase by 3 ± 1 mmHg compared with the saline study (Fig. 4C and D). Heart rate increased significantly in the GLP‐1 study by 7 ± 1 bpm, whereas heart rate remained constant in the saline study (Fig. 5G and H).

### Effects of GLP‐1 on subcutaneous, abdominal ATBF, leg blood flow, and splanchnic blood flow

**Figure 6:**
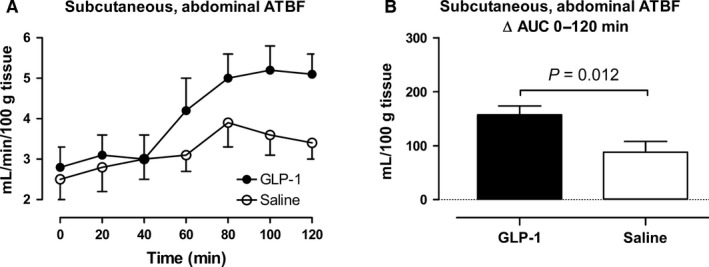
Subcutaneous, abdominal adipose tissue blood flow (adipose tissue blood flow) (A and B). A shows the time course of the measurements during the infusions from 0 to 120 min. B shows the integrated effect during the infusions from 0 to 120 min compared with baseline. Data are presented as means ± SE

**Figure 7:**
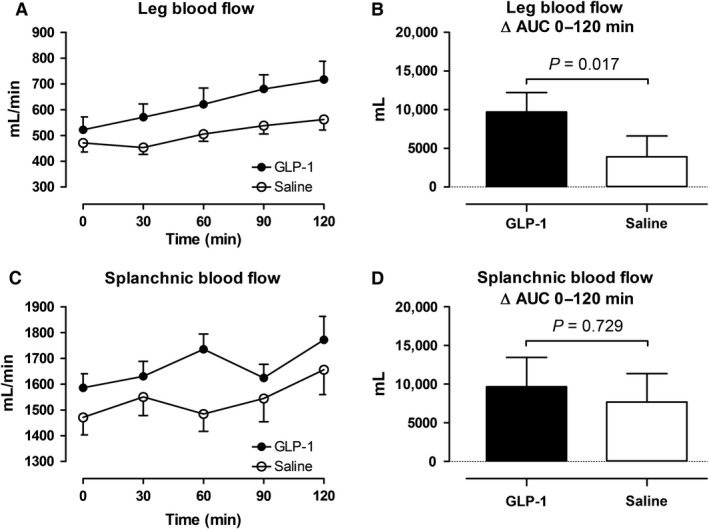
Leg blood flow (A and B) and splanchnic blood flow (C and D). Left panel shows the time course of the measurements during the infusions from 0 to 120 min. Right panel shows the integrated effect during the infusions from 0 to 120 min compared with baseline. Data are presented as means ± SE.

Subcutaneous, abdominal ATBF, leg blood flow, and splanchnic blood flow during the GLP‐1 and saline infusions are shown in Figure 6 and 7.

In the GLP‐1 study, subcutaneous abdominal ATBF began to increase after 40–60 min with a maximal increase (80–120 min after the commencement of the infusion) by 2.4 mL min^−1^ 100 g tissue^−1^. In the saline study, the peak increase (80–120 min after the commencement of the infusion) was 1.1 mL min^−1^100 g tissue ^−1^. In the GLP‐1 study, leg blood flow increased significantly to a maximal increase (90–120 min after the commencement of the infusion) by 195 mL min^−1^ compared to an increase by 83 mL min^−1^ in the saline study. In both studies, splanchnic blood flow did not change significantly, however, in the GLP‐1 study, splanchnic blood flow tended (*P* = 0.10) to increase initially (0–60 min after the commencement of the infusion) compared with the saline study.

## Discussion

In this study, we demonstrate that GLP‐1 elicits an increase in subcutaneous, abdominal ATBF during an acute administration of GLP‐1. Additionally, leg blood flow increases, whereas splanchnic blood flow remains unchanged. The increase in adipose tissue and skeletal muscle blood flow is consistent with most of the GLP‐1‐induced increase in cardiac output. Thus, we demonstrate a significant ~2‐fold sustained increase in subcutaneous, abdominal ATBF, 40–60 min after the commencement of the GLP‐1 infusion, which is a new in vivo observation. The increase in subcutaneous, abdominal ATBF was 2.4 mL min^−1^ 100 g tissue^−1^ during the last 60 min of the GLP‐1 infusion. If we assume that the GLP‐1‐induced increase in subcutaneous, abdominal ATBF is representative for the average body fat mass (14.8 kg, Table 1), it can be calculated that the increase in ATBF can account for ~35% of the increase in cardiac output in the last hour of the experiment.

Previously, only one study has investigated the effect of GLP‐1 on subcutaneous, abdominal ATBF.

Bertin et al. (2001), was not able to demonstrate any effect of locally infused GLP‐1 on subcutaneous, abdominal ATBF, using the microdialysis technique (ethanol inflow/outflow ratios). However, this technique is less sensitive compared with the ^133^Xe wash‐out technique to detect changes in ATBF (Karpe et al. 2002b). It is well described (Bulow et al. 1987a; Karpe et al. 2002a), that ATBF increases postprandially and that hyperinsulinemia per se cannot account for this increase (Asmar et al. 2010). Recently, we demonstrated in healthy lean subjects that glucose‐dependent insulinotropic polypeptide (GIP) increases subcutaneous, abdominal ATBF by ~5‐fold (Asmar et al. 2010, 2014, 2016b,c). However, the increase is dependent on postprandial hyperinsulinemia as well as hyperglycemia. In contrast to GIP, we demonstrated in this study, that GLP‐1 stimulates subcutaneous, abdominal ATBF at fasting glucose and insulin concentrations. Interestingly, the increase in subcutaneous, abdominal ATBF due to systemic exposure of GLP‐1 in this study is comparable to the 2–3‐fold increase in subcutaneous, abdominal ATBF, usually seen as a response to nutritional stimuli (Bulow et al. 1987a; Karpe et al. 2002a). Whether the effect of GLP‐1 may be potentiated by hyperglycemia and hyperinsulinemia, as is the case for GIP, needs to be studied in additional experiments. The fact, the GLP‐1′s vasodilatory effect in adipose tissue is delayed (by 20–40 min) compared to the GLP‐1‐induced vasodilation in skeletal muscle (discussed later) indicates that different mechanisms could be involved.

Hyperinsulinemia >400 pmol/L has in previous studies been shown to elicit increase in central sympathetic activity (Rowe et al. 1981; Vollenweider et al. 1993; Scherrer and Sartori 1997; Tack et al. 1998; Paolisso et al. 1999). However, in our previous study, conducted under the same experimental conditions as applied in this study, we were not able to measure any significant effect on plasma levels of noradrenaline or adrenaline. Additionally, heart rate increased similarly with no effect on heart rate variability. Altogether, indicating that a significant activation of the sympathetic nervous system was probably not induced in this study by the transient decrease in blood glucose levels induced by GLP‐1.

In a study by Hilsted et al. (1985), a decrease in subcutaneous ATBF was found during hypoglycemia (~2 mmol/L), induced by insulin injected intravenously. This vasoconstriction was probably due to stimulation of vascular *α*‐receptors by increased circulating catecholamines. In this study, blood glucose levels decreased transiently with a nadir of 4.36 ± 0.12 mmol/L from time 30–60 min after the commencement of the GLP‐1 infusion. In the same time interval (40–60 min), the subcutaneous, abdominal ATBF began to increase. It can be speculated that the mild hypoglycemia as seen in this study may have attenuated the initial GLP‐1‐induced vasodilation in the adipose tissue.

A sustained increase took place in leg blood flow during the GLP‐1 infusion. The increase in blood flow in the examined leg during the GLP‐1 infusion was 195 mL/min. Adipose tissue accounts for ~2.6 kg of the tissue mass in the examined leg (Table 1). Assuming that leg adipose tissue and subcutaneous, abdominal ATBF behave similarly postprandially as demonstrated previously (Manolopoulos et al. 2012), ~30% of the leg blood flow increase can be estimated to have taken place in adipose tissue. If we assume that the remaining increase in leg blood flow takes place in the skeletal muscles and that the skeletal muscles in the leg are representative for whole body skeletal muscles, this can account for ~40% of the observed increase in cardiac output (given that ~40% of total body weight is skeletal muscles).

There was a substantial arteriovenous difference in plasma levels of intact GLP‐1 across the lower extremity and splanchnic vascular bed, possibly reflecting expression of dipeptidyl peptidase‐4 (DPP‐4) in the endothelial membrane of the capillaries in these vascular beds. Interestingly, total GLP‐1, and thereby the metabolite GLP‐1 9‐36amide, which is not a substrate for DPP‐4 and if anything acts as an antagonist at the GLP‐1 receptor, was also cleared in the leg. Both intact GLP‐1 and its metabolite have previously (Drucker 2016) been reported to cause vasorelaxation, which was to some extent independent of the known GLP‐1 receptor. The mechanism responsible for the increased flow found here cannot be derived from the present results. Nevertheless, our findings are in accordance with previous studies, demonstrating an acute GLP‐1‐induced increase in flow‐mediated dilation of the brachial artery (Nystrom et al. 2004; Basu et al. 2007). Sjoberg et al. (2014) infused GLP‐1 (1.0 pmol kg^−1^ min^−1^) directly into the femoral artery in healthy subjects leading to supraphysiological plasma levels of GLP‐1 (20‐30‐fold increase compared with baseline). Independent of physiological hyperinsulinemia, this led to microvascular recruitment by ~60% in the vastus lateralis muscle of the infused leg after 5 min together with an increase in the diameter of the femoral artery by ~12%. In the noninfused contralateral leg, in which plasma levels of GLP‐1 was within the physiological range (7–10‐fold increase compared with baseline) the effect was slightly delayed but similar in magnitude. This is in accord with this study in which skeletal muscle blood flow (lower limb blood flow corrected for adipose tissue flow) increased during the GLP‐1 infusion, resulting in similar plasma levels of GLP‐1 as seen in the physiological part of the study by Sjoberg et al. (2014).

The splanchnic blood flow, measured by indocyanine green clearance technique, remained unaffected during the GLP‐1 infusion apart from a transient tendency to increase initially after the commencement of the GLP‐1 infusion. We could not demonstrate any significant splanchnic arteriovenous difference in plasma concentrations of total GLP‐1 or GLP‐1 9‐36amide. In a previous study, Trahair et al. (2014) demonstrated that under postprandial hyperglycemia and hyperinsulinemia (induced by a intraduodenal glucose infusion; 3 kcal/min) an intravenous infusion of GLP‐1 (0.9 pmol kg^−1^ min^−1^) increased blood flow in the superior mesenteric artery as measured by ultrasound/Doppler technique. Flow increased significantly more compared with the saline infusion under postprandial hyperglycemia and hyperinsulinemia. However, blood glucose concentrations were lower during the GLP‐infusion. Whether GLP‐1 has a role per se in the vasodilation in the splanchnic bed cannot be determined from the study by Trahair et al. (2014) since an oral glucose load initiates a similar vasodilatory effect (Bulow et al. 1999). An explanation for the constant splanchnic blood flow found in this study can be, that an increase in the portal vein blood flow has been counterbalanced by a vasoconstriction in the hepatic artery via the intrinsic autoregulation of the hepatic artery as well as the hepatic arterial buffer response (Eipel et al. 2010), a mechanism which previously has been demonstrated with respect to the splanchnic vascular effect of GIP (Kogire et al. 1988). However, in a recent human study, it has not been possible to demonstrate a GLP‐1‐induced vasodilation in the superior mesenteric artery by ultrasound/Doppler sonography technique (J.J. Holst, unpubl. data).

Together with our previous study, we have under similar conditions altogether examined the vascular biology of GLP‐1 in four major vascular beds; the renal, splanchnic, adipose tissue, and skeletal muscle. Considering the time course of blood flow changes in the examined vascular beds and the changes in cardiac output under slightly supraphysiological circulating GLP‐1 levels, the initial increase in cardiac output is likely due to an increase in skeletal muscle blood flow. Since GLP‐1 was extracted significantly in the lower extremity, this indicates that the vasodilation in the skeletal muscle may be elicited via GLP‐1 receptors (Pujadas and Drucker 2016). The later onset increase in ATBF, contributing to the GLP‐1‐induced increase in cardiac output may be due to a derived GLP‐1 effect. This mechanism needs to be elucidated in separate experiments.

### Limitations of the study

Firstly, the circulating levels of GLP‐1 outside the portal circulation are usually low due to a rapid degradation of GLP‐1 by DPP‐4. The slightly supraphysiological levels of GLP‐1 applied in this study may therefore not be completely translated into normal human physiological conditions. Secondly, the effect of acute elevation of plasma GLP‐1 concentrations for a few hours in a limited number of patients do not necessarily reflect the chronic elevation of GLP‐1 receptor agonist levels in a larger population with type 2 diabetes treated with a long‐acting GLP‐1 receptor agonist. Therefore, the existence of chronic effects that are not detected in the present experimental setup cannot be excluded.

### Conclusions

Under the conditions applied in the present experiments, acute administration of GLP‐1 increases blood flow in adipose tissue and skeletal muscle, whereas splanchnic blood flow remains unchanged. Together with an increase in cardiac work and thereby blood flow, this can explain the simultaneous increase in cardiac output in healthy lean subjects.

## Conflict of Interest

No conflicts of interest, financial or otherwise, are declared by the author(s).
